# Serum Irisin, Myostatin, and Myonectin Correlate with Metabolic Health Markers, Liver Disease Progression, and Blood Pressure in Patients with Metabolic Dysfunction-Associated Fatty Liver Disease and Hypertension

**DOI:** 10.3390/metabo14110584

**Published:** 2024-10-28

**Authors:** Anna F. Sheptulina, Elvira M. Mamutova, Anastasia Yu. Elkina, Yuriy S. Timofeev, Victoria A. Metelskaya, Anton R. Kiselev, Oxana M. Drapkina

**Affiliations:** 1 Department of Fundamental and Applied Aspects of Obesity, National Medical Research Center for Therapy and Preventive Medicine, 101990 Moscow, Russia; 2 Department of Therapy and Preventive Medicine, A.I. Yevdokimov Moscow State University of Medicine and Dentistry, 127473 Moscow, Russia; 3 Department of Intermediate Level Therapy, Saratov State Medical University, 410012 Saratov, Russia; 4 Coordinating Center for Fundamental Research, National Medical Research Center for Therapy and Preventive Medicine, 101990 Moscow, Russia

**Keywords:** metabolic dysfunction-associated steatotic liver disease, hypertension, metabolism, cardiometabolic risk, myokines

## Abstract

Background/Objectives: Recent data indicate the involvement of skeletal muscles in the regulation of metabolism and in the pathogenesis of chronic noncommunicable diseases. The goal of our study was to describe the serum concentrations of myokines in patients with metabolic dysfunction-associated steatotic liver disease (MASLD) and hypertension (HTN) and their correlation with laboratory parameters, blood pressure (BP), and MASLD severity. Methods: A total of 67 patients with MASLD and HTN underwent anthropometric measurements, laboratory tests, and point shear-wave elastography. The serum concentrations of myokines were measured using enzyme-linked immunosorbent assay (ELISA). Results: Patients with detectable serum myonectin concentrations had significantly higher maximum systolic blood pressure (*p* = 0.022) and higher blood levels of uric acid (*p* = 0.029). Serum irisin concentration ≥ 6.1 μg/mL was associated with higher FLI values (*p* = 0.042) and liver stiffness (*p* = 0.034), as well as with slightly higher waist circumference (*p* = 0.082) and triglyceride level (*p* = 0.062). Patients with serum myostatin concentration ≥ 4.98 ng/mL were significantly older (*p* = 0.033) and had a lower blood albumin level (*p* = 0.043). Conclusions: In conclusion, the myokine profile in patients with MASLD and HTN correlates both with the severity of MASLD and the parameters characteristic of metabolic health, suggesting the possible contribution of altered irisin, myonectin, and myostatin concentrations to the occurrence of cardiometabolic risks in patients with MASLD.

## 1. Background

Metabolic dysfunction-associated steatotic liver disease (MASLD), previously known as non-alcoholic fatty liver disease (NAFLD), is currently the most common chronic liver disease worldwide and is the leading indication for liver transplantation in the United States. This pathology has a variety of manifestations, described among all ethnic groups worldwide and present in both sexes [1]. MASLD encompasses a heterogeneous clinicopathological spectrum, ranging from fatty liver dystrophy to steatohepatitis and progressive liver fibrosis. The latter can lead to end-stage liver disease, such as cirrhosis and hepatocellular carcinoma.

Overall, the prevalence of MASLD has increased over the past 20 years. According to researchers, it is up to 40% in Western countries, while in the Asian world nearly 30% of the population suffers from this disease [2]. Prognostic models show that every third adult resident of the United States (i.e., more than 100 million adults) will suffer from MASLD by 2030 [3]. In Russia, its prevalence is similar to the global average: according to the multicenter observational study DIREG2, 37.3% of patients seeking outpatient care are diagnosed with MASLD [4].

MASLD is closely associated with obesity (especially abdominal obesity) and metabolic syndrome, which significantly increases cardiometabolic risk and affects morbidity, prognosis, and life expectancy in patients. For example, it was established that the presence of hypertension (HTN) in patients with MASLD, which is both a component of metabolic syndrome and a leading risk factor for cardiovascular disease, contributes to a significant exacerbation of the prognosis. Specifically, it increases the likelihood of MASLD progressing to cirrhosis and the development of hepatocellular carcinoma by 59% [5], along with an increase in the risk of developing cardiovascular diseases and cardiovascular mortality [6].

In obese patients, the prevalence of various clinical forms of MASLD is significantly higher than in the general population and, according to some studies, amounts to 75–93%, with NASH diagnosed in 18.5–26%, fibrosis in 20–37%, and cirrhosis in 9–10% of patients [7,8,9]. In addition, in patients with MASLD, despite excess body weight or obesity, a reduction in muscle strength and loss of muscle mass are often detected. This phenomenon is known as sarcopenic obesity. Therefore, this is a case of three important interrelated structures—that of liver–muscles–adipose tissue—the increasing dysfunction of which can lead to more severe pathological phenotypes and clinical outcomes. The negative effects of excess calories, physical inactivity, and genetic predisposition disrupt the regulatory pathways of energy metabolism, linking adipose tissue with skeletal muscle and the liver, which leads to adipose tissue remodeling with ectopic deposition of excess lipids. The latter contributes to lipotoxicity, inflammation, and insulin resistance, as well as resulting in negative effects on the intestinal microbiota, pancreatic β-cells, and, as a consequence, an increased risk of developing infections, cancer, and organ failure [10,11,12].

As research results have shown, skeletal muscles are not passive participants of the processes that occur in the human body during the development of MASLD [13]. They are capable of secreting biologically active substances known as myokines, the profile of which can change depending on the level of physical activity, the degree of fat infiltration in skeletal muscle, the amount of fat in the body, and a number of other factors [14,15,16]. Due to the fact that myokine receptors are located not only in the muscles per se, but also in the liver, adipose tissue, and pancreas, these compounds are able to participate in regulating the functioning of the aforementioned organs [15,17,18]. In particular, it is well known that myokine receptors are expressed by stellate cells of the liver responsible for the synthesis of the extracellular matrix and the development of fibrosis [19,20].

In addition, myokines can regulate total metabolism by influencing the metabolism of glucose and lipids, since disruption of these processes is the basis for the pathogenesis of MASLD [17,18]. Besides the fact that increased physical activity remains to this day the only effective way to prevent the development and slow the progression of MASLD—and that muscle contraction is precisely the process by which myokines such as myonectin and irisin are produced by muscles [21,22]—it is possible that at least some of the beneficial effects of physical activity on metabolism and the course of MASLD are associated with the action of myokines.

The musculoskeletal system has long remained outside the focus of attention of doctors and researchers investigating MASLD; however, as recent studies have demonstrated, skeletal muscles can be considered an endocrine organ capable of synthesizing biologically active substances involved in the metabolism and regulation of body composition, thereby determining the likelihood of forming conditions for the development of obesity, MASLD, and various other chronic diseases. For instance, it has been demonstrated that myostatin may be independently associated with the development and progression of atherosclerosis [23]. In contrast, J. Lu et al. showed that irisin appears to have an endothelium-protective action, which might be explained by the activation of the adenosine monophosphate (AMP)-activated protein kinase-phosphatidylinositol 3-kinase-Akt-endothelial nitric oxide synthase (AMPK-PI3K-Akt-eNOS) signaling pathway [24]. In turn, in their study, M. Maciorkowska et al. demonstrated that irisin was capable of reducing blood pressure via a nitric oxide-dependent pathway. In addition, it was shown that patients with type 2 diabetes mellitus (T2D) had lower serum irisin levels compared to non-diabetic individuals, thus indicating irisin’s role in regulating glucose metabolism [25]. Finally, a study by A. Leiherer et al. suggests that plasma myonectin levels are significantly associated with T2D in elderly cardiovascular disease patients [26]. Taken together, these conditions constitute cardiometabolic risk factors, and according to recently published criteria [27], the presence of at least one such factor is required to establish the diagnosis of MASLD. Regarding MASLD itself, it has been demonstrated that serum irisin levels correlate with the severity of liver inflammation [28] and may contribute to liver fibrogenesis [29]. At the same time, low plasma myostatin levels reduce the expression of proinflammatory cytokines involved in the development of steatohepatitis and are associated with improved insulin sensitivity [30,31].

Thus, taking all the aforementioned into consideration, we conducted a study aimed at investigating the serum profile of myokines (myonectin, myostatin, and irisin) in patients with MASLD and HTN, and evaluated the correlations between serum concentrations of myokines, biochemical parameters indicative of metabolic health, liver disease severity, and HTN grade in this patient category.

## 2. Materials and Methods

### 2.1. Patients

**Figure 1:**
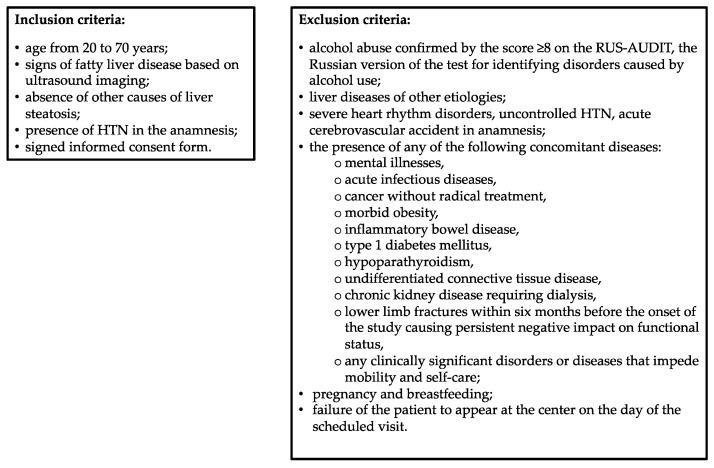
Inclusion and exclusion criteria. HTN, hypertension; RUS-AUDIT, Russian Alcohol Use Disorders Identification Test.

This cross-sectional single-center study included patients with HTN and MASLD aged 20 to 70 years who were seeking medical assistance at the Department of Clinical Diagnostics of the National Medical Research Center for Therapy and Preventive Medicine of the Russian Federation Ministry of Healthcare in the period from February 2023 through April 2024, met the inclusion criteria, and did not comply with the exclusion criteria. The study protocol was approved by the local Ethics Committee (protocol No. 01-03/20 of 23 January 2020). All study participants signed written informed consent prior to their inclusion in the study. The inclusion and exclusion criteria are shown in Figure 1.

All procedures required for the study, including anamnesis compilation, physical examination, anthropometric measurements—such as weight, height, body mass index (BMI), waist circumference (WC), and hip circumference (HC)—laboratory tests (including complete blood count, blood chemistry and myokine profile), and abdominal ultrasound imaging, were performed during a single patient visit to the center.

### 2.2. Diagnoses of Metabolic Dysfunction-Associated Steatotic Liver Disease and Hypertension

The diagnosis of MASLD was established based on the results of ultrasound imaging of the abdominal organs, specifically the presence of signs of hepatic steatosis (increased echogenicity of the liver in comparison with the renal cortex and/or depletion of vascularization and/or attenuation of the ultrasound signal along the periphery of the organ). A mandatory criterion for inclusion in the study was the presence of HTN in the patient, which is a known cardiometabolic risk factor. It is worth noting that the presence of at least one such factor is an essential condition for the diagnosis of MASLD according to an expert consensus published in 2023 [27]. Thus, the diagnosis of MASLD in this study was established based on the detection of signs of hepatic steatosis and the presence of HTN as a cardiometabolic risk factor. Patients with serum ALT activity greater than 33 U/l (males) or 25 U/l (females) underwent additional testing to exclude other causes of hepatic steatosis (markers of hepatitis B and C viruses) [32].

In addition, to assess the likelihood of hepatic steatosis, the fatty liver index (FLI) was calculated as follows [33]:

FLI = [e^0.953×ln(TG)+0.139×BMI+0.718×ln(GGT)+0.053×WC−15.745^/(1 + e^0.953ln(TG)+0.139×BMI+0.718×ln(GGT)+0.053×WC−15.745^)] × 100,

where TG stands for triglycerides and GGT is an acronym for gamma-glutamyl transferase.

An FLI value less than 30 (negative likelihood ratio = 0.2) allowed us to exclude hepatic steatosis, whereas a FLI value of 60 or more (positive likelihood ratio = 4.3) confirmed the presence of hepatic steatosis [33].

We also performed liver stiffness measurement using point shear-wave elastography. At least ten valid measurements were included in the final report. We considered liver stiffness assessment reliable if the following criteria were met: 10 valid measurements, success rate > 60%, and the ratio of interquartile range to median (IQR/M) ≤ 30% [34,35].

The diagnosis of HTN was established on the basis of anamnestic data and available medical documentation, and classified as grade 1 HTN with systolic blood pressure (SBP) values of 140–159 mmHg and/or diastolic blood pressure (DBP) of 90–99 mmHg; grade 2 HTN with SBP values of 160–179 mmHg and/or DBP of 100–109 mmHg; and grade 3 HTN with SBP values ≥ 180 mmHg and/or DBP ≥ 110 mmHg [36].

### 2.3. Assessment of Anthropometric Indicators

Height was measured using a R-St-MSK stadiometer (MSK-234, Medstalkonstruktsiya LLC, Ufa, Russia) via the conventional method. Body weight was measured using the electronic floor medical scales placed on a flat smooth floor (VMEN-200-50/100-I-ST-A, TVES LLC, Tambov Oblast, Russia). The BMI was calculated as body weight divided by height squared (kg/m^2^).

WC and HC were measured on the standing patient with the stomach relaxed, arms loosely lowered along the body, and heels together. WC was measured with a tape at the level of the narrowest part of the abdomen (i.e., at the level of the natural waist) at the end of a normal exhalation, pressing the tape to the clothes but without pressing it into the skin. When measuring HC, the tape was placed around the hips at the level of the maximum protrusion of the buttocks.

### 2.4. Blood Myokine Profile Study

The determination of erythroferrone (myonectin), myostatin, and irisin (FNDC5, fibronectin type III domain-containing protein 5) levels was performed in blood serum collected using the standard method on an empty stomach, processed according to the recommendations of The Biospecimen Reporting for Improved Study Quality (BRISQ), and kept in a secure, reliable storage at a temperature from −70 °C to −80 °C in the Bank of Biological Material of the National Medical Research Center for Therapy and Preventive Medicine.

The quantitative determination of biochemical markers in blood serum was carried out by direct solid-phase enzyme-linked immunosorbent assay (ELISA) using standardized test systems.

Erythroferrone was determined using the ELISA kit for Erythroferrone EFRE (Cloud-Clone Corp., Wuhan, China/ Katy, TX, USA) with a measurement range of up to 10.0 ng/mL (10,000 pg/mL) and an analytical sensitivity of 0.064 ng/mL (64 pg/mL).

For the analysis of serum myostatin concentrations, we used the ELISA kit for Myostatin (MSTN) by Cloud-Clone Corp., Wuhan, China/USA with a calibration range of 0.625–40 ng/mL (analytical sensitivity of 0.237 ng/mL).

Irisin was determined in the dilutions recommended by the reagent manufacturer with phosphate-buffered saline (PBS, 0.01 mmol/L, pH 7.0–7.2) using the ELISA kit for fibronectin type III domain-containing protein 5 FNDC5 (Cloud-Clone Corp., Wuhan, China/USA) with an analytical sensitivity of 5.5 pg/mL.

Serum samples and reagents were dispensed using dispensers that had passed the necessary metrological tests. A PST-60HL plate shaker-thermostat (Biosan/VectorBest, Riga/Moscow, Latvia/RF) was used to incubate the microplates, maintaining a temperature of 37.0 °C according to the test system manufacturer’s instructions. For microplates, we used an automatic microplate washer, Anthos Fluido (Biochrom, Cambridge, UK).

Optical density (absorbance) was measured using a Multiskan FC microplate photometer (Thermo Fisher Scientific, Waltham, MA, USA) at a wavelength of 450 nm with a reference wavelength of 620 nm (metrological verification of November 2023). Calibration curves were plotted and concentrations were calculated using arigo’s ELISA GainData calculator (ArigoBio, Zhubei City, Hsinchu County, Taiwan; https://www.arigobio.com/elisa-calculator accessed on 2 September 2024).

### 2.5. Statistical Analyses

The Kolmogorov–Smirnov test was employed to analyze the distribution type. Absolute values and proportions (percentage of the total) are given for categorical variables. Numerical data are presented as median and interquartile range (IQR, 25th; 75th percentiles) due to non-compliance with the normal distribution. Spearman’s correlation coefficient (ρ) was used to analyze the degree of relationship between two variables. The strength of the correlation was assessed according to the Chaddock scale as negligible (0.1 ≤ ρ < 0.3), weak (0.3 ≤ ρ < 0.5), moderate (0.5 ≤ ρ < 0.7), strong (0.7 ≤ ρ < 0.9), and very strong (0.9 ≤ ρ < 1.0) [37]. Comparison of two groups by numerical variables was performed using the Mann-Whitney U test, while the nonparametric Kruskal–Wallis test was employed to compare several groups by numerical variables. Pearson’s chi-squared test was used to compare groups by categorical variables. The significance level for all tested hypotheses was accepted at *p* < 0.05. Statistical data processing was performed using the IBM SPSS software version 27.0 (IBM Corp., Armonk, NY, USA).

## 3. Results

### 3.1. General Characteristics of Patients

**Figure 2:**
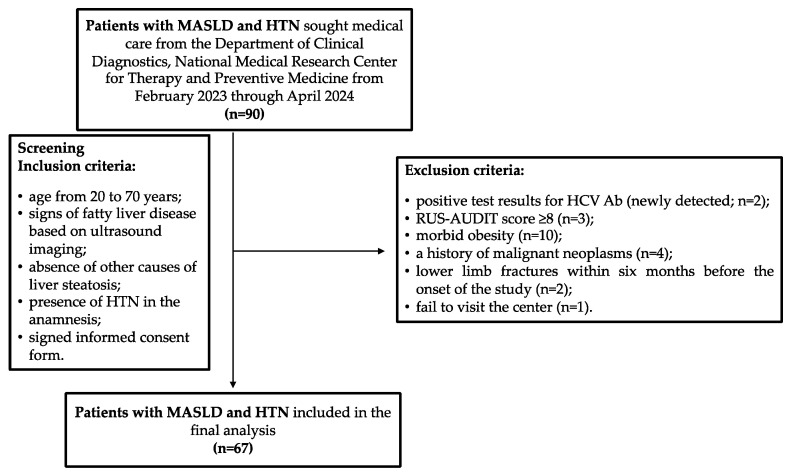
Participants flow chart. HCV Ab, antibodies to hepatitis C virus; HTN, hypertension; MASLD, metabolic dysfunction-associated steatotic liver disease; RUS-AUDIT, the Russian Alcohol Use Disorders Identification Test.

In total, from February 2023 through April 2024, 90 patients with established diagnoses of MASLD and HTN who met the inclusion criteria for the study sought help from the Department of Clinical Diagnostics, National Medical Research Center for Therapy and Preventive Medicine. However, during the screening procedure, the following exclusion criteria were identified in 23 patients: 2 patients had positive test results for antibodies to the hepatitis C virus (HCV Ab, newly detected); 3 patients had a RUS-AUDIT score ≥ 8; 10 patients had morbid obesity; 4 patients had a history of malignant neoplasms, which was revealed during their conversation with a doctor (2 patients had a history of breast cancer; 1 patient had colon cancer and 1 patient had kidney cancer); in 2 patients, there were health issues qualifying as exclusion criteria 2 and 3 months before inclusion in the study, respectively; and 1 patient did not show up at the center on the scheduled appointment day and the study physicians could not contact him by phone or email (Figure 2). Thus, 67 patients were included in the final analysis, and their main characteristics are presented in Table 1. The median age of the patients was 58 years (IQR: 51–65 years). Obesity was detected in 52 patients (65.7%), and 2 patients (2.9%, both female) had BMI values within the normal range, but their WC values were greater than 80 cm (91 and 94 cm), indicating cardiometabolic risk.

Twenty-two (32.8%) patients were diagnosed with grade 1 HTN, 22 (32.8%) patients with grade 2 HTN, and 23 (34.3%) patients with grade 3 HTN. No statistically significant differences in gender and age were revealed between patients with grades 1, 2, or 3 HTN. T2D was present in 7 (10.4%) patients, of which 4 (57.1%) were women with a median age of 59 years (IQR: 51–65 years), and impaired fasting glycemia (IFG) was detected in 9 (13.4%) patients, of which 7 (77.8%) were women with a median age of 57 years (IQR: 52–67.5 years). Patients with T2D or IFG did not significantly differ statistically from patients without these conditions in terms of their gender or age. Table 2 presents the main results of the laboratory tests performed on the patients included in the study.

Antihypertensive therapy was received by 49 (73.1%) patients included in the study, of which 33 were female. Its characteristics are presented in Table 3.

Combination antihypertensive therapy typically included a combination of a diuretic and a renin-angiotensin-aldosterone system (RAAS) blocker (n = 17, 54.8%), a calcium channel blocker and a RAAS blocker (n = 4, 12.9%), a beta blocker and a calcium channel blocker (n = 5, 16.1%), or a beta blocker and a RAAS blocker (n = 5, 16.1%).

### 3.2. Assessment of Myokine Concentrations in the Blood Serum of Patients with Metabolic Dysfunction-Associated Steatotic Liver Disease and Hypertension

The results of the study of myokine content in the blood serum of patients with MASLD and HTN are presented in Table 4.

Myonectin was detected in the blood serum of 22 patients (32.8%). Their median age was 61 years (IQR: 45.8–65.5 years), and 12 of the 22 patients (54.5%) were female. Half of the patients (11, 50%) suffered from grade 3 HTN. T2D was diagnosed in 1 patient (4.5%), while 4 patients (18.2%) had IFG. According to these parameter values, as well as BMI values, patients with MASLD and HTN in whom myonectin was detected in the serum did not significantly differ from the group of patients with MASLD and HTN in whom myonectin was not detected in the serum. The FLI values and the measured liver stiffness based on point-shear wave elastography data were similar between the groups of patients with MASLD and HTN with detectable and undetectable serum myonectin concentrations.

**Figure 3:**
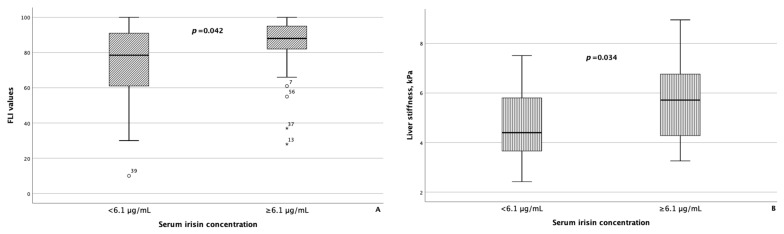
Fatty liver index values (**A**), as well as liver stiffness (**B**), were significantly higher in patients with MASLD and HTN with serum irisin concentrations ≥ 6.1 μg/mL compared with patients with MASLD and HTN with serum irisin concentrations less than 6.1 μg/mL The line through the middle of each box represents the median. The height of each box represents the interquartile range. The whiskers show the minimum and maximum values of each subscale. Outliers are depicted as circles and asterisks. All comparisons were performed using the Mann–Whitney U test. FLI: Fatty liver index.

As reference values for serum myokine concentrations are currently lacking, to compare the groups of patients with MASLD and HTN depending on the serum concentrations of myostatin and irisin, the modes of serum concentrations of myostatin and irisin, which were 4.98 ng/mL and 6.1 μg/mL, respectively, were set as the threshold values for these parameters. In 37 of 67 (55.2%) patients with MASLD and HTN, the concentration of myostatin was less than 4.98 ng/mL, and in 31 of 67 (46.3%) patients with MASLD and HTN, the concentration of irisin was less than 6.1 μg/mL. The characteristics of the groups of patients with MASLD and HTN depending on the concentrations of myostatin and irisin in the blood serum are presented in Table 5. Patients with a concentration of myostatin in the blood serum of less than 4.98 ng/mL and ≥4.98 ng/mL did not differ statistically significantly in terms of FLI values and liver stiffness based on the point-shear wave elastography data. At the same time, in patients with the concentration of irisin in the blood serum ≥ 6.1 μg/mL, the FLI value and the result of liver elastography were statistically significantly higher than in the group of patients with MASLD and HTN with the concentration of irisin in the blood serum less than 6.1 μg/mL (*p* = 0.042 and *p* = 0.034, respectively) (Figure 3).

The correlation analysis established an inverse correlation between the serum concentration of myostatin and ALT activity (ρ = −0.279, *p* = 0.023), along with a direct correlation between the serum concentration of myonectin and the blood level of uric acid (ρ = 0.273, *p* = 0.029). The serum concentration of irisin significantly correlated with the uric acid level in the blood (ρ = 0.238, *p* = 0.01), and also with the FLI value (ρ = 0.290, *p* = 0.026).

**Figure 4:**
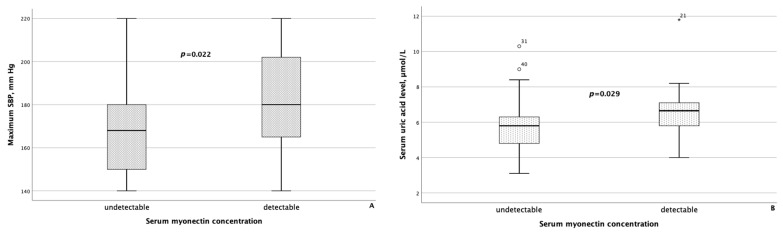
Maximum systolic blood pressure (**A**) and serum uric acid levels (**B**) were significantly higher in patients with MASLD and HTN with detectable myonectin concentrations. The line through the middle of each box represents the median. The height of each box represents the interquartile range. The whiskers show the minimum and maximum values of each subscale. Outliers are depicted as circles and asterisks. All comparisons were performed using the Mann–Whitney U test. SBP: systolic blood pressure.

When comparing clinical and laboratory data with body composition parameters between groups of MASLD patients differing in serum concentrations of myokines, the following results were obtained. Patients with detectable myonectin concentrations had statistically significantly higher maximum SBP (*p* = 0.022) and higher serum uric acid levels (*p* = 0.029), as shown in Figure 4.

**Figure 5:**
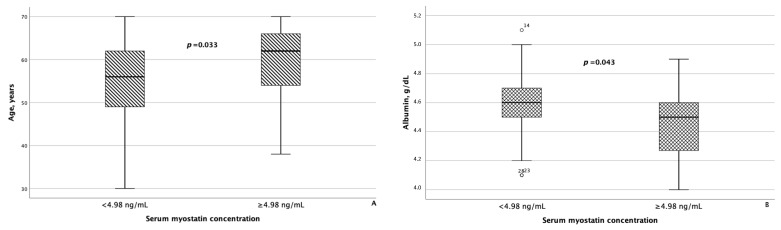
Patients with MASLD and HTN with serum myostatin concentrations ≥ 4.98 ng/mL were significantly older (**A**) and had significantly lower serum albumin levels (**B**) compared with patients with MASLD and HTN with serum myostatin concentrations < 4.98 ng/mL. The line through the middle of each box represents the median. The height of each box represents the interquartile range. The whiskers show the minimum and maximum values of each subscale. Outliers are depicted as circles. All comparisons were performed using the Mann–Whitney U test.

As for irisin, in addition to statistically significant differences in FLI and liver stiffness between the groups of patients with MASLD and HTN with serum irisin concentrations < 6.1 μg/mL and ≥6.1 μg/mL, minor differences were also confirmed in WC (*p* = 0.082) and blood triglyceride levels (*p* = 0.062). Besides age, patients with MASLD and HTN with serum myostatin concentrations < 4.98 ng/mL and ≥4.98 ng/mL differed significantly from each other with respect to blood albumin levels (*p* = 0.043), as seen in Figure 5. In addition, patients with serum myostatin concentrations ≥ 4.98 ng/mL had slightly lower liver stiffness compared with patients with MASLD and HTN with serum myostatin concentrations < 4.98 ng/mL (*p* = 0.062).

## 4. Discussion

Currently, MASLD is the most common chronic liver disease among adults and children worldwide [2]. Although most patients have simple steatosis with no obvious symptoms, in 50% of cases the disease can progress to steatohepatitis, cirrhosis, or hepatocellular carcinoma [1]. Starting recently, it has become common to talk about the multisystem nature of MASLD, implying that this liver disease is associated with a high risk of developing various chronic noncommunicable diseases [38]. This concept is based on the idea of the unity of pathogenetic mechanisms and risk factors for MASLD, along with diseases of other organs and systems, one of which may be the musculoskeletal system [10]. It has been shown that due to the secretion of biologically active substances by the liver, skeletal muscles, bones, and adipose tissue (hepatokines, myokines, osteokines, and adipokines), these organs and tissues can interact with each other, transmitting signals and regulating physiological and pathological processes [39].

The goal of this cross-sectional single-center study was to investigate the serum concentrations of myokines (irisin, myonectin, and myostatin) in the patients with MASLD and HTN (as a cardiometabolic risk factor, the presence of which is a prerequisite for the diagnosis of MASLD, according to the expert consensus published in 2023 [27]) and to assess their correlation with laboratory parameters that are surrogate markers of metabolic disorders and liver disease activity [40,41,42].

According to the obtained data, patients with MASLD and HTN whose serum irisin concentrations did not exceed 6.1 μg/mL had lower FLI and liver stiffness values, compared with patients with serum irisin concentrations ≥ 6.1 μg/mL. These data are consistent with the results of the study by S.A. Polyzos et al., who demonstrated a direct correlation between the serum level of irisin and severe inflammation in the liver tissue of patients with MASLD. The authors concluded that the elevated level of irisin in this case may be a manifestation of a compensatory mechanism aimed at inducing an anti-inflammatory response [28]. Our data were also confirmed by S. Petta et al., who showed that irisin expression correlated with the severity of MASLD and the formation of extracellular matrices in the liver. They concluded that irisin participated in the processes of fibrogenesis in the liver. In particular, they showed that activated hepatic stellate cells were capable of synthesizing irisin, and in MASLD patients with fibrosis corresponding to the F2–F4 grades on the METAVIR scale, higher concentrations of irisin in the blood serum were observed [29].

A recently published study showed that FLI values ≥ 60 used to diagnose MASLD are also a marker of high cardiometabolic risk in a patient, which is partially influenced by insulin resistance, accumulation of abdominal fat, elevated blood pressure, and hyperlipidemia [43]. These data are confirmed by the results of J.H. Roh et al., who showed that higher FLI values were independently associated with a high risk of HTN in healthy individuals [44]. All factors listed in the study by F. Carli et al. [43] were present in patients with MASLD and HTN included in the present study. In addition, a systematic review by J.D.S. Pinho-Jr., which included 30 articles published between 2013 and 2020, confirmed that irisin can act both as a target for prevention and as a biomarker for the presence of obesity-associated comorbidities and cardiometabolic disorders [45].

Our study also described a trend towards increased WC and blood triglyceride levels in patients with MASLD and HTN with serum irisin concentrations ≥ 6.1 μg/mL. These data are supported by the results of previously published studies [46,47,48] and can be explained for several reasons. First, along with skeletal muscles, adipose tissue is also involved in the production of irisin; hence, its concentration may be higher in obese patients. Another explanation is that in patients who are overweight and/or obese, the amount of muscle in the body may be increased due to excess weight, which also contributes to the growth of serum irisin concentration [49,50]. In addition, in the study by M.K. Nilofer Sagana et al., an inverse relationship was demonstrated between the blood level of irisin and the value of the triglyceride glucose index in individuals aged 18 to 35 years. This finding allowed the authors to conclude that these markers can be used to identify dyslipidemia associated with a high risk of developing metabolic disorders and atherosclerosis [51].

Patients with detectable serum myonectin levels had higher SBP and higher uric acid levels vs. patients with MASLD and HTN and undetectable serum myonectin. Some studies have suggested that myonectin can activate insulin signaling pathways in adipose tissue and skeletal muscle, thereby improving glucose uptake in these tissues [14]. Its action is similar to that of adiponectin, and adipokine known for its anti-inflammatory effect and ability to increase insulin sensitivity. Z.M. Ismail et al. demonstrated that patients with T2D and nephropathy exhibited statistically significantly lower serum myonectin levels vs. the control group and patients with T2D but without nephropathy. Besides that, it was shown that the level of myonectin in the blood serum of patients with T2D and no nephropathy was statistically significantly higher than that of the control group [52]. These data are consistent with the results of the 2018 study by Z. Li et al., who demonstrated that myonectin concentrations in the blood serum of patients with newly diagnosed T2D and patients with impaired glucose tolerance (IGT) significantly exceeded those in healthy individuals. Moreover, they were higher in patients with T2D compared with individuals with IGT. In this study by Z. Li et al., a direct correlation was found between plasma myonectin levels and the following body composition parameters and biochemical parameters: waist-to-hip ratio, body fat percentage, blood triglyceride levels, fasting blood glucose, 2-h glucose tolerance test, fasting blood insulin levels, glycosylated hemoglobin (HbA1c), and HOMA-IR. At the same time, plasma myonectin levels were inversely related to the insulin sensitivity index. These patterns were observed in all study participants [53]. It is possible that an increase in myonectin concentration is a coping strategy, driven with the steadily increasing concentration of lipids in the blood and gradually filling fat depots against the background of the progression of metabolic disorders. Since uric acid level indicates an adverse metabolic condition, and HTN is considered a component of metabolic syndrome, the association between serum myonectin concentrations and the listed parameters determined in our study further supports the idea that serum myonectin concentrations (in particular, its increase in serum) may act as a marker of metabolic disorders and the body’s attempts to cope with them.

According to our results, serum myostatin concentration increased with increasing age in patients with MASLD. These data are consistent with the results obtained by B.R. McKay et al., who showed that the level of myostatin in the blood serum and the level of myostatin mRNA in the muscles were twofold in elderly men (70 ± 4 years) compared with young control subjects (21 ± 3 years) [54]. It is the colocalization of myostatin and stem cells in the muscles (myosatellite cells) of elderly individuals that is the consequence of the impaired ability of muscles to regenerate and of the sarcopenia development.

The inverse relationship between serum concentrations of albumin and myostatin observed in our study was also described in the publication by E. Yasar et al., which included patients with different stages of chronic kidney disease [55]. In the study by T. Alexopoulos et al., it was shown that a model incorporating the concentrations of myostatin, creatine phosphokinase, and albumin had a high accuracy in excluding the presence of sarcopenia in patients with cirrhosis [56]. Similarly, H. Nishikawa et al. discovered that serum albumin concentration, as well as psoas muscle index and prothrombin time, were inversely related to blood myostatin levels in patients with cirrhosis. The authors suggested that higher blood levels of myostatin in patients with cirrhosis were associated with muscle loss, hyperammonemia, and impaired protein synthesis, which was reflected in low serum albumin concentration [57]. The results of the aforementioned studies are consistent with our findings regarding higher myostatin concentrations in individuals with lower liver stiffness values.

## 5. Limitations of the Study

This study has a number of limitations. One of them is the small sample size. Nonetheless, the patients selected for the study constituted a representative group of patients with MASLD without significant liver fibrosis who underwent a comprehensive examination. Therefore, the patterns identified in this study can be extrapolated to all patients with MASLD. Another limitation of the study is the absence of a control group. However, the study design and objectives involved describing the myokine profile in patients with MASLD in combination with such cardiometabolic risk factors as HTN, as well as analyzing its relationship with clinical data and laboratory test results. Our study design did not involve comparing the myokine profiles between patients with MASLD and healthy individuals. Certainly, a comparative study would provide more data on changes in the myokine profile in patients with MASLD vs. healthy controls. One more limitation of the study is that we did not use liver biopsies to diagnose MASLD or determine the degree of disease activity and stage of fibrosis. For these purposes, we used the methods recommended by the European Association for the Study of the Liver (EASL) guidelines: ultrasound imaging of the liver according to the standard protocol and liver elastography [58].

## 6. Conclusions

The results of our study indicate the existence of a relationship between serum myokine concentrations, laboratory parameters characterizing metabolic health, indicators of liver disease progression, as well as BP values in patients with MASLD and HTN, thereby confirming the assumption of the role of myokines in the regulation of metabolism and in the occurrence of cardiometabolic risks in patients with MASLD. The study of the myokine profile in MASLD, as well as in other MASLD-associated diseases, is valuable not only from the standpoint of searching for noninvasive markers of the disease and its progression, but also from the standpoint of identifying new potential therapeutic targets and factors reflecting the dynamics of changes during treatment.

## Figures and Tables

**Table 1:** Demographic and anthropometric parameters of patients with MASLD and HTN included in the study.

Parameter	Patients with MASLD and HTN (*n* = 67)
Gender: female, n%	43 (64.2)
Age, years	58 (51–65)
BMI, kg/m^2^	33.2 (30.4–36.9)
Normal weight (18.5 ≤ BMI < 25 kg/m^2^), n (%)	2 (2.9)
Overweight (25 ≤ BMI < 30 kg/m^2^)	13 (19.4)
Obesity (BMI ≥ 30 kg/m^2^)	52 (77.6)
Waist circumference (all subjects), cm	106.5 (102–113.2)
Waist circumference (male), cm	108 (106–116.5)
Waist circumference (female), cm	106 (100–112.3)
Hip circumference, cm	114 (109–119)

Note: For numerical data, median and interquartile range are presented; for categorical variables, absolute values and percentages are presented; BMI, body mass index; HTN, hypertension; MASLD, metabolic dysfunction-associated steatotic liver disease.

**Table 2:** Results of laboratory tests in patients with MASLD and HTN included in the study.

Parameter	Patients with MASLD and HTN (*n* = 67)
Erythrocytes, 10^12^/L	4.7 (4.41–4.9)
Hemoglobin, g/L	145 (133–151)
ESR, mm/h	9 (4–14)
Platelets, 10^9^/L	245 (210–286)
Leukocytes, 10^9^/L	6.1 (5.3–7.2)
ALT, IU/L	23 (17–38)
AST, IU/L	22 (18–26)
GGT, IU/L	31.5 (21–46.3)
Albumin, g/dL	4.5 (4.4–4.7)
Total bilirubin, μmol/L	12 (9–15)
Uric acid, μmol/L	6 (5.3–7)
CRP, mg/L	2.7 (1.4–5.5)
Total cholesterol, mmol/L	5.8 (5.1–6.2)
Triglycerides, mmol/L	1.43 (1.04–2.04)
Creatinine, μmol/L	74 (68–88)
Glucose, mmol/L	5.7 (5.4–6.1)
HOMA-IR > 2.7, n (%)	47 (70.1)
Liver stiffness, kPa (pSWE)	5.1 (4.1–6.3)

Note: For numerical data, median and interquartile range are presented; for categorical variables, absolute values and percentages are presented. ALT, alanine aminotransferase; AST, aspartate aminotransferase; CRP, C-reactive protein; ESR, erythrocyte sedimentation rate; GGT, gamma-glutamyl transferase; HOMA-IR, Homeostasis Model Assessment of Insulin Resistance; HTN, hypertension; MASLD, metabolic dysfunction-associated steatotic liver disease; pSWE, point-shear wave elastography.

**Table 3:** Characteristics of the antihypertensive therapy received by patients in the study.

Category of Pharmaceutical Drugs	Number of Patients with MASLD and HTN, *n* (%)
Calcium channel blockers	1 (1.5)
Beta blockers	4 (5.9)
Angiotensin receptor blockers	8 (11.8)
ACE inhibitors	5 (7.4)
Combination antihypertensive therapy	31 (45.6)

Note: ACE, angiotensin-converting enzyme; HTN, hypertension; MASLD, metabolic dysfunction-associated steatotic liver disease.

**Table 4:** Serum concentrations of irisin, myonectin, and myostatin in patients with MASLD and HTN (n = 67).

Myokine	Median Concentration	IQR
Irisin (all patients), µg/mL	6.04	5.1–7.3
Irisin (men), µg/mL	6.1	5.7–8.1
Irisin (women), µg/mL	6.0	4.49–7.2
Myonectin (all patients), pg/mL	0.0	0.0–78.5
Myonectin (men), pg/mL	0.0	0.0–82.8
Myonectin (women), pg/mL	0.0	0.0–75.3
Myostatin (all patients), ng/mL	4.6	3.4–5.8
Myostatin (men), ng/mL	4.2	2.9–5.7
Myostatin (women), ng/mL	4.9	3.6–5.9

Note: HTN, hypertension; IQR, interquartile range; MASLD, metabolic dysfunction-associated steatotic liver disease.

**Table 5:** Characteristics of groups of patients with MASLD and HTN depending on the concentrations of myostatin and irisin in the blood serum.

Parameter	Serum Irisin Concentration in Patients with MASLD and HTN, μg/mL			Serum Myostatin Concentration in Patients with MASLD and HTN, ng/mL		
	<6.1 (n = 31)	≥6.1 (n = 30)	*p*	<4.98 (n = 37)	≥4.98 (n = 30)	*p*
Age, years	59 (53–64)	60 (49–66)	n/s	56 (47.5–62.5)	62 (53.5–66)	0.033
Gender: female, n (%)	22 (70.9)	18 (60)	n/s	22 (59.5)	20 (66.7)	n/s
BMI, kg/m^2^	33 (30.8–36.9)	33.9 (29.5–37.2)	n/s	33.1 (30.8–36.7)	33.6 (30.1–36.9)	n/s
HTN, n (%):			n/s			n/s
Grade 1	11 (35.5)	8 (26.7)		11 (29.7)	10 (33.3)	
Grade 2	11 (35.5)	9 (30)		13 (35.1)	9 (30)	
Grade 3	9 (29)	13 (43.3)		13 (35.1)	11 (36.7)	
T2D, n (%)	5 (16.1)	2 (6.7)	n/s	5 (13.5)	2 (6.7)	n/s
IFG, n (%)	7 (22.6)	2 (6.7)	n/s	4 (10.8)	5 (16.7)	n/s

Note: For numeric variables, median and interquartile range are presented; for categorical variables, absolute values and percentages are provided; comparisons between numeric variables were performed using the Mann–Whitney U test; comparisons between categorical variables were performed using the chi-squared test. n/s, the differences are not statistically significant; BMI, body mass index; HTN, hypertension; IFG, impaired fasting glycaemia; MASLD, metabolic dysfunction-associated steatotic liver disease; T2D, type 2 diabetes mellitus.

## Data Availability

The data presented in this study are available on reasonable request from the corresponding authors. The data are not publicly available due to privacy restrictions.
